# Crystal Structure of the Multidomain Pectin Methylesterase PmeC5 from *Butyrivibrio fibrisolvens* D1^T^

**DOI:** 10.3390/biom15050720

**Published:** 2025-05-14

**Authors:** Vincenzo Carbone, Kerri Reilly, Carrie Sang, Linley R. Schofield, William J. Kelly, Ron S. Ronimus, Graeme T. Attwood, Nikola Palevich

**Affiliations:** AgResearch Limited, Grasslands Research Centre, Palmerston North 4442, New Zealand

**Keywords:** pectin methylesterase, *Butyrivibrio*, rumen, pectin, methanol

## Abstract

Pectin is a dynamic and complex polysaccharide that forms a substantial proportion of the primary plant cell wall and middle lamella of forage ingested by grazing ruminants. Pectin methylesterases (PMEs) are enzymes that belongs to the carbohydrate esterase family 8 (CE8) and catalyze the demethylesterification of pectin, a key polysaccharide in cell walls. Here we present the crystal structure of the catalytic domain of PmeC5 that is associated with a gene from *Butyrivibrio fibrisolvens* D1^T^ that encodes a large secreted pectinesterase family protein (2089 aa) determined to a resolution of 1.33 Å. Protein in silico modelling of the secreted pectinesterase confirmed the presence of an additional pectate lyase (PL9) and adhesin-like domains. The structure of PmeC5 was the characteristic right-handed parallel β-helical topology and active site residues of Asp231, Asp253, and Arg326 typical of the enzyme class. PmeC5 is a large modular enzyme that is characteristic of rumen *B. fibrisolvens* megaplasmids and plays a central role in degrading plant cell wall components and releasing methanol in the rumen environment. Such secreted PMEs are significant contributors to plant fiber digestion and methane production, making them attractive targets for both methane mitigation strategies and livestock productivity enhancement.

## 1. Introduction

In the rumen environment, methanol (CH_3_OH) is primarily generated through the demethylation of pectin, a complex polysaccharide found in plant cell walls. This process is catalyzed by the enzyme pectin methylesterase (PME), which hydrolyzes the methyl ester bonds of pectin, liberating methoxy groups (-OCH_3_). Subsequently, specialized methanogenic archaea, known as methylotrophic methanogens, utilize the released methanol as a substrate for methanogenesis. These microorganisms employ a metabolic pathway that reduces methanol to methane (CH_4_) through a series of enzymatic reactions that contribute to the overall production of enteric methane in ruminants, a potent greenhouse gas. Species within the genus *Butyrivibrio* are prominent pectin-degrading bacteria in the rumen, playing a critical role in the hydrolysis of pectin and contributing to CH_3_OH production. In addition to their involvement in methanol formation, these bacteria are integral to fiber degradation, protein digestion, and the biohydrogenation of unsaturated fatty acids. Given that methanol release during pectin degradation serves as a substrate for methanogenic archaea, targeting this process represents a potential strategy for methane (CH_4_) mitigation in ruminants.

The fermentative catabolism of organic matter in the rumen ecosystem generates various metabolic end products, including dihydrogen (H_2_), carbon dioxide (CO_2_), and C_1_ compounds such as methanol (CH_3_OH), methylamines (R-NH-CH_3_), and methylsulphides (R-S-CH_3_). While these compounds are not directly utilized by the host animal for nutritional purposes, they serve as crucial electron acceptors and carbon sources for specialized methanogenic archaea, particularly methylotrophic methanogens [1,2]. These microorganisms employ these substrates in their energy metabolism, utilizing them in redox reactions that culminate in the production of methane (CH_4_) [3]. This process not only represents a significant pathway for electron disposal in the anaerobic rumen environment but also contributes substantially to enteric methane emissions from ruminant livestock [4,5].

Emerging hypotheses propose that enteric methane (CH_4_) production in ruminants could be mitigated by limiting the availability of methylotrophic substrates (e.g., methanol, methylamines) within the rumen environment [6,7]. Specifically, depleting the methanol reservoir generated via microbial degradation of plant-derived compounds such as pectin would directly impair the metabolic activity of methylotrophic methanogens, which rely on these C_1_ substrates for energy conservation via methanogenesis. Such substrate limitation strategies, potentially achieved through targeted manipulation of pectin degradation pathways or enzymatic inhibition of methyl-group liberation, may disrupt this archaeal metabolic niche. The activity and regulation of PMEs are therefore crucial for understanding plant cell wall digestibility, rumen fermentation dynamics, and potential strategies for mitigating enteric methane production in ruminant livestock.

Plant cell walls are primarily composed of cellulose, hemicellulose, xylan, lignin, and pectin, which together provide structural integrity and functionality [8]. Pectin represents a structurally complex and heterogeneous group of polysaccharides that play a multifaceted role in plant biology [9]. It contributes to the regulation of cell wall architecture and expansion, mediates cell-cell adhesion and communication, and participates in signaling pathways [10]. Additionally, pectin is a key component in plant defense mechanisms, acting as a barrier against pathogens and facilitating the activation of immune responses [11].

Pectin, a complex heteropolysaccharide, is predominantly localized in the middle lamella of the primary cell wall. It comprises two major structural domains: homogalacturonan (HG) and rhamnogalacturonan I (RG-I), with minor components including xylogalacturonan (XGA), arabinan, arabinogalactan I, and rhamnogalacturonan II (RG-II) [10]. The structural backbone of pectin is primarily composed of α-(1,4)-linked D-galacturonic acid residues, not β-(1,4)-D-galactan. This backbone is diversely substituted with side chains containing galacturonic acid, rhamnose, xylose, and arabinose residues [12]. The degree of substitution and the presence of acetyl and methyl groups on the galacturonic acid residues are highly variable, contributing to the structural complexity and functional diversity of pectin in plant cell walls [13]. Pectin methylesterases (PMEs, EC 3.1.1.11, classified in the Carbohydrate Esterase family 8 [CE8]) are crucial enzymes that modulate the physicochemical properties and digestibility of plant cell wall components [14]. These enzymes catalyze the de-esterification of the C_6_-linked methyl esters of galacturonic acid residues in the homogalacturonan regions of pectin [15]. This hydrolytic process results in the liberation of methanol (CH_3_OH) and the formation of free carboxyl groups on the pectin backbone. In the context of ruminant digestion, the methanol produced through PME activity becomes a significant carbon and energy source for specialized methylotrophic methanogens [16]. These archaea utilize methanol in their energy metabolism, reducing it to methane (CH_4_) through a series of enzymatic reactions [1]. This process not only contributes to the overall methane emissions from ruminants but also represents an important pathway for hydrogen disposal in the anaerobic rumen environment.

Members of the genus *Butyrivibrio* serve as keystone rumen microbiota [17], mediating three critical processes: (i) plant fiber deconstruction via lignocellulolytic activity [18,19], (ii) proteolytic digestion of dietary proteins [20], and (iii) biohydrogenation of unsaturated fatty acids [21,22]. Additionally, they contribute substantially to ruminal methanol production through pectin demethylation [23,24,25]. The type strain *Butyrivibrio fibrisolvens* D1^T^ [26] exhibits specialized pectinolytic capabilities and encodes a suite of carbohydrate-active enzymes (CAZymes) to depolymerize pectin into galacturonate, arabinose, and galactose [18,26,27,28]. These monomers undergo fermentation via the 2-keto-3-deoxygluconate (KDG) pathway, yielding butyrate, formate, and acetate as primary metabolic end products [18]. Genomic analyses reveal *B. fibrisolvens* D1^T^ encodes expansive polysaccharide utilization loci (PULs) specific for pectin [26]. These CAZy families include glycoside hydrolases (GH28 for polygalacturonase activity), polysaccharide lyase (PL9 targeting rhamnogalacturonan I/II), and carbohydrate esterases (CE12 for acetyl group removal; CE8 pectin methylesterases [PMEs]) [29,30].

PME genes encoding CE8 protein domains are conserved across multiple strains of *B. fibrisolvens*, though their abundance and specific genomic configurations vary [18]. These PMEs can be categorized into intracellular and extracellular types, each with distinct roles, localizations, and implications for rumen function. Intracellular *Butyrivibrio* PMEs may support bacterial growth by metabolizing smaller partially degraded pectic oligogalacturonates or modifying pectic intermediates that are transported into bacterial cells for energy production [29]. *B. fibrisolvens* and *B. proteoclasticus* intracellular PMEs have recently been characterized and are not directly involved in plant fiber degradation [30]. In contrast, extracellular PMEs directly initiate degradation of plant cell wall components, particularly homogalacturonan of pectin, releasing methanol and exposing galacturonic acid residues as described above. Importantly, rumen *B. fibrisolvens* strains also possess PME genes that encode large multidomain secreted proteins (approximately 2000–2200 amino acids in size) combining a PL9 family pectate lyase with cell wall-binding domains [18]. Pectate lyases (PL9) also play a vital role in pectin degradation by cleaving the α-(1→4)-linked galacturonic acid in homogalacturonan via β-elimination, generating 4,5-unsaturated oligogalacturonates. Moreover, these large multidomain PMEs encode an N-terminal signal peptide sequence that directs extracellular secretion along with several cell wall-binding domains or likely carbohydrate-binding modules (CBMs) that anchor the enzyme to the plant cell wall components. These additional domains are proposed to cooperatively enhance substrate accessibility by facilitating direct interactions with pectin substrates, suggesting a specialized role of these large multidomain enzymes in plant cell wall modification or degradation.

*B. fibrisolvens* also exemplify evolutionary adaptation for plant cell wall deconstruction and complete pectin breakdown in the rumen with their multi-replicon genome architecture [26]. The complete genome of D1^T^ consists of a 4,671,138 bp circular chromosome, a small 21 Kb plasmid (pNP21), and a 243 Kb megaplasmid (pNP243). The later pNP243 secondary replicon encodes genes for glycosyl transferases, hydrogenases (*echABCDEF*, *hypA*, and *hypFCDE* maturation proteins), and the PmeC5 with a modular domain architecture reported in this study. Interestingly, the co-location of similar large extracellular PMEs and other genomic components associated with the metabolism of extracellular polysaccharides appears to be a feature of all rumen *B. fibrisolvens* strains.

From a biotechnological perspective, *B. fibrisolvens* and both their intracellular and extracellular PMEs have potential uses in biofuel production (e.g., methanol and hydrogen), industrial food processing, and feed digestibility due to their ability to modify complex polysaccharides and improve nutrient availability for ruminants. Moreover, targeted inhibition of CE8 PMEs could disrupt the pectin → CH_3_OH → CH_4_ pipeline with a number of strategies under investigation, including: (i) small-molecule inhibitors blocking PME active sites [31,32], (ii) phage-derived peptides interfering with PUL assembly, and (iii) CRISPR-Cas9 editing of CE8 genes in rumen microbiomes [33]. Such enzymatic targeting approaches are appealing as they are proposed to minimize collateral damage to non-pectinolytic rumen microbiota, thus preserving fiber degradation capacity while specifically curbing methanol-driven methanogenesis. To guide future targeted inhibition studies, we have elucidated the crystal structure and active site for PmeC5 from *B. fibrisolvens* D1^T^.

## 2. Materials and Methods

### 2.1. Molecular Modelling

The expression profiles over time of *Butyrivibrio* PMEs associated with methanol release have been previously identified [6] and modelled in Carbone et al. 2023 to confirm that PmeC5 is indeed a pectin methylesterase (Enzyme Classification 3.1.1.11). PmeC5 was modelled using the online tool AlphaFold2 version 2.3.1 [34] for comparison and used when carrying out molecular replacement to solve the crystallographic structure. Additional enzymes were modelled utilizing the online platform Chai-1 [35] and analyzed against published structures using Dali [36]. The targeted enzymes were visualized and figures generated using PyMOL Molecular Graphics System version 2.0 (Schrödinger version 4.6).

Molecular docking was carried out using the program GOLD (Genetic Optimization for Ligand Docking) version 5.1 [37] and the crystal structure of PmeC5. Given the inherent flexibility of loops and their positioning within this enzyme class, an ensemble docking protocol was carried out alongside PmeC5 with the pectin methylesterase structure of 2NTP and its bound hexasaccharide VI substrate [38]. This enabled the validation of the in silico docking protocol and the identification and potential positioning of substrate within the active site of PmeC5. The in silico docking protocol favored the GOLD Fitness function and incorporated all residues that fell within 6 Å of the hexasaccharide VI substrate of 2NTP and the superimposed PmeC5 structure. A 100% search efficiency was employed, generating ten genetic algorithm runs for the docked molecule, and all sidechain residues remained rigid. The generated binding poses and scores were inspected, with conformations chosen for further analysis by taking into account their ranking and interactions with the active site residues.

### 2.2. Protein Expression and Purification

PmeC5 catalytic domain protein purification utilized steps previously described for similar bacterial pectin methylesterases in Carbone et al. 2023 [30]. The final concentration of the enzyme was 3.6 mg/mL, in storage buffer containing 20 mM 3-(*N*-morpholino)propanesulfonic acid (MOPS) pH 7.0 and 2-mM beta-mercaptoethanol (BME).

### 2.3. Crystallization

The crystallization condition for PmeC5 (3.6 mg/mL) was identified using the Molecular Dimensions (UK) Shot Gun screen (SG1) and the sitting drop method on 96-well 2 Drop UV crystallization plates. Crystals grew over several weeks in mother liquor containing 0.2 M ammonium chloride and 20% (*w*/*v*) PEG 3350 and were cryo-protected in mother liquor containing 25% (*v*/*v*) ethylene glycol prior to freezing in liquid nitrogen.

### 2.4. Data Collection and Structure Determination

Diffraction data were collected at the Australian Synchrotron MX1 beamline at 100° K and processed with XDS and Aimless 0.7.15 [39,40]. Total exposure time was 72 s for a single 360-degree oscillation of the crystal at a detector distance of 130 mm. Beam attenuation was also adjusted to optimize the collection of data to a resolution of 1.33 Å. PmeC5 crystallized in the monoclinic crystal system P12_1_1 with unit cell parameters a = 43.30 Å, b = 61.95 Å, c = 77.37 Å, α = 90.00°, β = 105.39°, and γ = 90.00°, with a single monomer in the asymmetric unit and a solvent content estimated to occupy 41.25% of the unit cell volume. Initial phases of the enzyme were determined by the molecular replacement program MOLREP version 11.0 [41] using the AlphaFold2 model [34]. All structural idealization was carried out utilizing repeated cycles of restrained refinement in REFMAC 5.8.0425 [42]. Weighted difference-Fourier maps (2Fo-Fc and Fo-Fc) were visualized following each cycle in Coot 0.9.8.95 [43] to enable the rebuilding of loops and sidechains and the addition of water and other associated molecules present in the crystallization matrix. Structural coordinates have been deposited under accession code 9MM2.

## 3. Results and Discussion

### 3.1. The Pectin Methylesterase Pmec5 Structure from B. Fibrisolvens D1^T^

**Figure 1:**
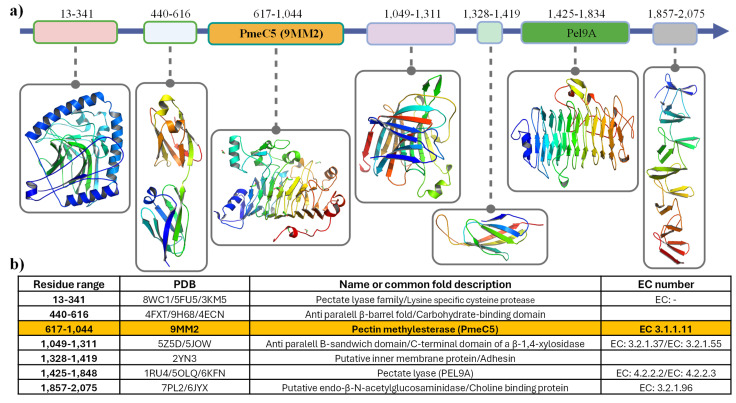
Predicted structural domains of the multidomain pectin methylesterase from *Butyrivibrio fibrisolvens* D1^T^. (**a**) Multidomain structure of the rumen bacterial pectin methylesterase family protein from *B. fibrisolvens* D1^T^ (WP_073390447.1). (**b**) Structural matches for the modelled putative protein homologs are shown, discovered using a PDB50-based Dali alignment.

The 427 amino acid sequence of the PmeC5 catalytic domain (pfam01095) from *B. fibrisolvens* D1^T^ is associated with a gene that encodes a large secreted pectinesterase family protein (2089 aa, locus tag WP_073390447). This gene encodes several other protein domains with RefSeq annotations, including a domain of unknown function (DUF5018, pfam16410), pectin methylesterase and related acyl-CoA thioesterase (PemB, COG4677), autotransporter adhesin (AidA, COG3468), and glucan-binding domain (YG repeat, COG5263). Additional annotation methods have also detected a Pel9A-like, right-handed beta helix region that corresponds to a PL9 family pectate lyase domain (pfam22842). To confirm the identity and location of these domains, we modelled the enzymes utilizing the online platform Chai-1 and compared them to published structures using Dali (Figure 1). Our results coincide with the assertion of the presence of Pel9A beta-helical domain, adhesin-like domains, and our own pectin methylesterase.

**Figure 2:**
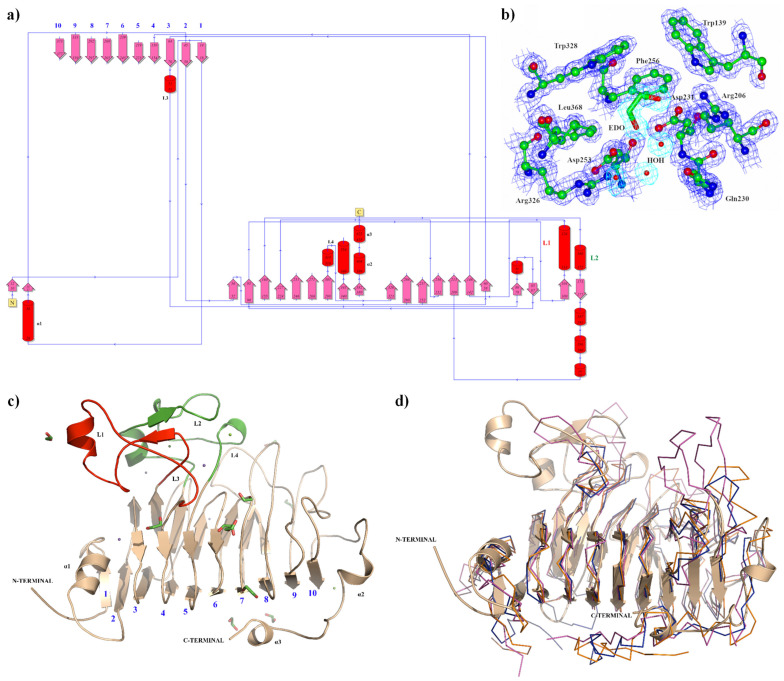
The crystal structure of PmeC5 (9MM2) from *B. fibrisolvens* D1^T^. (**a**) Illustrates the protein’s topology in terms of how the β-strands (pink arrows) are arranged into β-sheets, the position of α-helices (red cylinders), and their respective residue numbers. (**b**) Depicts the active site domain and corresponding electron density (2Fo-Fc) in blue for the amino acid sidechains and in aqua marine for the solute molecules ethylene glycol (EDO) and water (HOH). (**c**) A ribbon representation of the PmeC5 monomer (light brown). The N- and C-terminal domains are labelled, as well as loop 1 (L1 in red), loop 2 (L2 in green), loop 3, and loop 4 (L3 and L4). The 10 beta sheets that form right-handed parallel β-helical topology are numbered. (**d**) The crystal structure of PmeC5 in ribbon form (light brown) superimposed with the unique members of the larger PME enzyme superfamily (EC 3.1.1.11) discovered during PDB50-based Dali [44] alignment, including 5C1C from *Aspergillus niger* (dark blue), 1QJV PemA from *Erwinia chrysanthemi* (purple), and the plant pectin methylesterase 1GQ8 from *Daucus carota* (orange).

The apo crystal structure of the PmeC5 catalytic domain monomer was determined to a maximum resolution of 1.33 Å, displaying well-resolved and continuous electron density for the observable mainchain and sidechains. The full data collection and refinement statistics for PmeC5 are listed in Table 1. This excluded the N-terminal residues Met1 through to Ser8 with no discernible electron density. Molecular replacement was carried out using an AlphaFold2 model of PmeC5, with the structures producing near identical secondary structural arrangement (0.388 Å RMSD). This included a number of elongated loops, with the most prominent being an N-terminal positioned alpha helical bundle and small anti-parallel beta sheet formed by residues 96–138 (L1) and residues 160–206 (L2), as shown in Figure 2. This extends from the structurally distinctive right-handed parallel β-helical topology common to all PMEs [44,45].

This β-helical structure is formed by a 10-turn parallel β-sheet (Figure 2), and, as seen in some recent bacterial PME structures [30], they are in possession of several elongated loops connecting them in addition to L1 and L2. This includes a third N-terminal loop (L3 residues 70–89) toward the C-terminal L4 (residues 303–321, Figure 2). The N-terminal region of PmeC5 is composed of a short α-helix (α1) followed by a β-strand that aligns with and helps form the 10-turn parallel β-sheet, and the C-terminus comprises a single long loop (residues 378–423) containing two short α-helices (α2; residues 399–403, α3; residues 418–421). The overall structure of PmeC5 is maintained by an internal aligned core of hydrophobic amino acids (Ile16, Ile47, Leu67, Phe150/Ala152, Phe221, Phe243, Phe263, Met295, and Phe337) placed at equivalent positions on neighboring β-strands throughout its helical topology (Figure 2).

### 3.2. The Substrate Binding Domain of Pmec5

**Figure 3:**
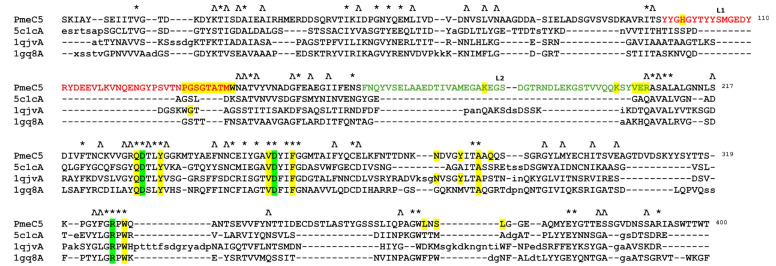
The PDB50-based Dali [46] structural alignment of the bacterial pectin methylesterase PmeC5. Identical amino acid residues are marked with a *, with near identical residues with a Ʌ. Structural gaps are shown with a –, while insertions are shown in lower case. Catalytic residues Asp231, Asp253, and Arg326 are highlighted in green. Potential substrate binding residues His99, Pro131, Gly132, Ser133, Gly134, Thr135, Ala136, Thr137, Met138, Trp139, Asn140, Lys181, Lys201, Val204, Glu205, Arg206, Gln230, Tyr234, Val252, Phe256, Asn277, Tyr281, Ala284, Gln286, Trp328, Leu365, Ser367, and Leu368 are highlighted in yellow. Residue numbering is based on PmeC5.

**Figure 4:**
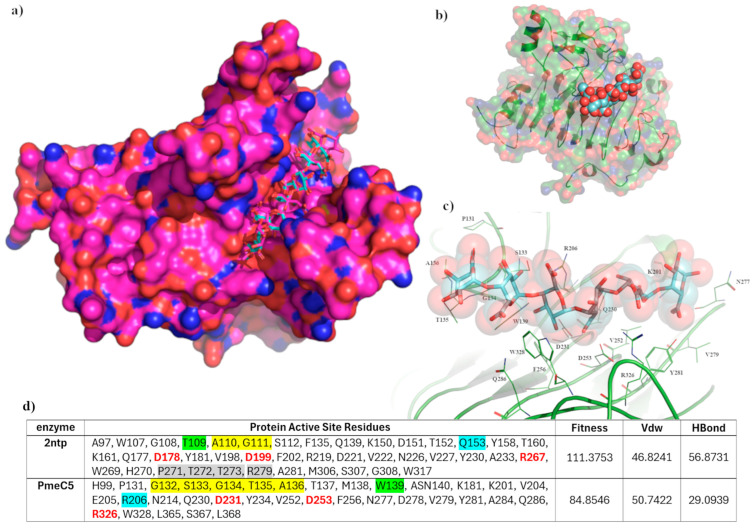
In silico docking results. (**a**) A validation of the docking protocol, with the superimposed top-ranked hexasaccharide VI substrate (in blue) docked into the active site of 2NTP. (**b**) The relative positioning of the pectin binding domain of PmeC5, measuring approximately 25.1 Å long and 9.4 Å wide. (**c**) The catalytic center of PmeC5, with the docked hexasaccharide VI substrate (in blue sticks and residues within 4 Å of the substrate as thin green lines) formed by catalytic residues Asp231, Asp253, and Arg326. (**d**) Active sites considered during in silico docking and scoring for the hexasaccharide VI substrate. Significant residues in identical positions are color-coded, with red being the catalytic residues and yellow-, green-, and blue-highlighted residues directly contributing to the observed differences in pectin binding and orientation.

The catalytic and active site residues of PmeC5 were first delineated using the online Dali-based PDB50 structural alignment [46] program (where enzymes are sorted by a sequence identity of less than fifty per cent of PmeC5) and further extrapolated using in silico docking of a potential pectin substrate (Table 2, Figure 3 and Figure 4). The catalytic triad consisting of an arginine and two aspartic acid residues is strictly conserved amongst PMEs [30] and is formed by Asp231, Asp253, and Arg326 in PmeC5. Each residue is immediately adjacent to one another on beta turns 6, 7, and 9 (and the L1 and L2 loops of PmeC5), and they are all largely solvent accessible, as seen on similar members of the larger PME enzyme superfamily (EC 3.1.1.11) discovered during Dali alignment (Figure 3). These enzymes include the fungal PME 5C1C from *Aspergillus niger* (RMSD 2.1 Å) [47], PemA 1QJV from *Erwinia chrysanthemi* (RMSD 2.4 Å) [42], the plant pectin methylesterase 1GQ8 from *Daucus carota* (RMSD 2.4 Å) [48], and the bacterial acyl-CoA thioester hydrolase 3GRH from *Escherichia coli* (RMSD 2.4 Å) [49]. Superimposition of the enzymes (Figure 2) shows that the larger active site of the molecules, in addition to the cleft formed by the catalytic residues, is formed in part and bordered by loops of differing lengths and positions between beta sheets, as shown by the many sequence gaps within the structural alignment (Figure 3). As such, we estimate the pectin-binding domain of PmeC5 to measure approximately 25.1 × 9.4 Å.

The pectinesterase of 2NTP [38] possesses the same basic fold structure as PmeC5, and its bound hexasaccharide VI substrate allows us to distinguish the larger substrate binding domain of PmeC5. Utilizing the GOLD software program [37] and ensemble docking mode, we were able to validate a docking algorithm that mimicked the pose of the hexasaccharide VI substrate in 2NTP (Figure 4). The top-ranked model closely mimicked the molecular interactions of the crystal structure, with the largest deviations seen in the solvent-exposed saccharide units. Seven of the top ten generated binding poses favoured 2NTP, while the binding poses for PmeC5 were positionally similar overall (Figure 4b,c), for the first three D-galactopyranuronic acid moieties. However, the terminal methyl-α-D-galactopyranuronate significantly deviates due to the positioning of the L1 loop (residues 131–136), in particular Gly134 and Thr135, occupying and directly overlapping the region in which the sugar orients on an elongated loop, which is completely absent in 2NTP.

Immediately adjacent to this pocket, additional, non-conserved amino acid residues of 2NTP also contributed to the change of positioning, including Thr272 and Arg279 present on a small β-sheet (residues 272–281) known to contribute to the preference for a carboxylate rather than a methylester substrate [36]. In addition, the exchange of residues Trp139/Thr109 and Gln153/Arg206 for 2NTP and PmeC5, respectively, downstream of the catalytic center (Figure 4d) also affects the substrate positioning and presumably substrate preference. Scoring was also consistently lower for PmeC5 w.r.t hydrogen bonding and van der Waals interactions. Overall, in the apo form of PmeC5, the additional pectin or substrate binding residues as scored by GOLD during in silico docking would include Pro131, Ser133, Gly134, Thr135, Ala136, Trp139, Lys201, Arg206, Gln230, Val252, Phe256, Asn277, Val279, Tyr281, Gln286, Trp328, and Leu368.

## 4. Conclusions

Rumen *Butyrivibrio fibrisolvens* demonstrate significant biotechnological potential due to their genomic plasticity and expansive repertoire of extracellular polysaccharide-degrading enzymes. In this study, the crystal structure of the pectin methylesterase PmeC5 catalytic domain from the rumen bacterium *B. fibrisolvens* D1^T^ has been elucidated. The structure of this catalytic domain reveals a right-handed β-helical structure with relatively simple loops forming the walls of the active site to create a relatively shallow catalytic groove when compared with other PME enzymes. Elucidation of the structural components of the multimodal *B. fibrisolvens* pectin esterases (e.g., PmeC5) provides critical insights for designing effective inhibitors and advancing efforts to reduce greenhouse gas emissions without compromising biomass conversion efficiency. Pectin methylesterases have the potential for broad biotechnological applications, such as biofuel production (particularly methanol and hydrogen generation), food processing, and enhancing ruminant feed digestibility through polysaccharide modification. The clear preference for producing these multimodal enzymes in the rumen environment remains to be unravelled, although it may reflect biologically relevant functionalities related to improving nutrient accessibility by initiating the breakdown of complex plant biomass. For future work, we aim to characterize the molecular mechanisms driving pectin breakdown, with potential applications in the food, textile, and feed industries, as well as methane mitigation by targeting methanol formation in the rumen.

## Figures and Tables

**Table 1:** Data collection and refinement statistics for PmeC5 (9MM2).

Data Collection Statistics *	Items
Space group	P12_1_1
Unit cell parameters: a, b, c (Å) α, β, γ (°)	43.30, 61.95, 77.37 90.00, 105.39, 90.00
Wavelength (Å)	0.953722
Temperature (K)	100
Resolution Range (Å)	47.66–1.33
No. of observed ref.	624,009 (30,348)
No. of unique ref.	89,955 (4440)
R_sym_ ^a^	0.045 (0.274)
R_pim_ ^b^	0.028 (0.171)
Completeness (%)	99.4 (98.4)
Multiplicity	6.9 (6.8)
I/σ(I)	23.3 (5.8)
*CC*_1/2_	1.000 (0.958)
**Refinement statistics**	
Resolution range (Å)	47.66–1.33
All reflections used	90,509
Size R_free_ set (%)	5
All reflections (R_free_)	4472
**R-values**	
R_cryst_ (%)	11.97
R_free_ (%)	14.01
Matthews coefficient (Å^3^ Da^−1^)	1.74
Solvent content (%)	28.70
RMSD **	
Rms Bond Length (Å)	0.0127
Rms Bond Angle (°)	1.8987
**Ramachandran plot**	
Residues in favored regions (%)	96.9
Residues in allowed regions (%)	3.1
**Average B factors** (**Å^2^**)	
Protein	9.055
Water (HOH)	23.282
Mg^2+^ Na	20.333 18.173

* Data in the highest resolution shell are given in parentheses (1.35–1.33 Å). ref., reflections; ** RMSD, root mean square deviation. ^a^ $R_{sym}=\frac{\sum_{hkl}\sum_{j}\left|I_{hkl,j}-\langle I_{hkl}\rangle \right|}{\sum_{hkl}\sum_{j}I_{hkl,j}}\;$. ^b^ R_pim_ denotes precision, indicating merging R-factor value $R_{pim}=\frac{\sum_{hkl\;}\sqrt{\frac{1}{n-1}}\;\sum_{j=1}^{n}\;|I_{hkl,j}-\langle I_{hkl}\rangle |\;}{\sum_{hkl}\sum_{j}I_{hkl,j}}$.

**Table 2:** Structural matches for bacterial pectin methylesterase PmeC5 using a PDB50-based Dali alignment. The *Dickeya dadantii* structure 2NTP is included for reference.

Organism and Gene	Class	PDB Monomer	Z-Score ^a^	RMSD ^b^	Lali ^c^	%id ^d^
*Dickeya dadantii* 3937 Gene Name: *PEMA*, *PEM* EC: 3.1.1.11	Pectinesterase	2NTP-A	33.2	2.4	289	27
*Aspergillus niger ATCC 1015* Gene Name: *ASPNIDRAFT_214857* EC: 3.1.1.11	Pectinesterase	5C1C-A	32.7	2.1	271	30
*Erwinia chrysanthemi* Gene Name: *PEMA* EC: 3.1.1.11	Pectin methylesterase	1QJV-A	32.7	2.4	290	27
*Daucus carota* Protein sequence: *P83218*, *PME* EC: 3.1.1.11	Pectinesterase	1GQ8-A	31.5	2.4	284	27
*Escherichia coli K-12* Gene Names: *b0772*, *JW0755*, *ybhC* EC: 3.1.2	Acyl-CoA thioester hydrolase	3GRH-A	28.9	2.4	269	19
*Sitophilus oryzae* Gene Name: *CE8-1* EC: 3.1.1.11	Pectinesterase	4PMH-A	28.6	2.2	261	26
*Butyrivibrio fibrisolvens* Gene Name: *SAMN02745229_01989* EC: 3.1.1.11	Pectinesterase	8TMS-A	25.0	2.9	229	22

^a^ A measure of the statistical significance of the result relative to an alignment of random structures. ^b^ Root mean square deviation (RMSD) of alpha-carbon atoms. ^c^ Number of aligned residues. ^d^ Sequence identity between the two chains.

## Data Availability

The structure coordinates and reflection files are deposited in the Protein Data Bank under accession number 9MM2.
